# *Plasmodium vivax* CSP-Pvs25 variants from southern Mexico produce distinct patterns of infectivity for *Anopheles albimanus versus An. pseudopunctipennis*, in each case independent of geographical origin

**DOI:** 10.1186/s13071-019-3331-0

**Published:** 2019-02-20

**Authors:** Lilia González-Cerón, Mario H. Rodríguez, José A. Nettel-Cruz, Juan E. Hernández-Ávila, Iliana R. Malo-García, Frida Santillán-Valenzuela, Cuauhtémoc Villarreal-Treviño

**Affiliations:** 1 Centro Regional de Investigación en Salud Pública, Instituto Nacional de Salud Pública, Tapachula, 30700 Chiapas México; 2 Centro de Investigación en Enfermedades Infecciosas, Instituto Nacional de Salud Pública, Cuernavaca, 62100 Morelos México; 3 Centro de Información para Decisiones en Salud Pública, Instituto Nacional de Salud Pública, Ciudad de México, 14080 México

**Keywords:** *Anopheles albimanus*, *Anopheles pseudopunctipennis*, Susceptibility, *Plasmodium vivax*, Mexico, Guatemala, Circumsporozoite, Pvs25, Gametocytes

## Abstract

**Background:**

The susceptibility of *Anopheles albimanus* and *An. pseudopunctipennis* to local *Plasmodium vivax* has been associated in southern Mexico with two ookinete surface proteins (Pvs25/28) polymorphism. Perhaps parasite population selection (i.e. adaptation to local vectors) contributes to this phenomenon. It is also possible that certain molecular interactions exist between *P. vivax* and each mosquito species independently of geographical origin. This study aimed to explore the susceptibility of *An. albimanus* and *An. pseudopunctipennis* (collected from different geographical sites) to *P. vivax* cspVk/Pvs25-130 haplotypes from southern Mexico.

**Results:**

Of the 120 *P. vivax*-infected blood samples used to simultaneously feed *An. albimanus* and *An. pseudopunctipennis* mosquitoes originating from various geographical sites, 80 produced at least one infected mosquito species. Three parasite haplotypes were identified in infected blood: Vk210/Pvs25-A (12.5%), Vk210/Pvs25-B (20%) and Vk247/Pvs25-B (67.5%). Two parameters (the proportion of infected mosquitoes and number of oocysts/mosquito) showed a similar pattern for each mosquito species (independently of geographical origin). For *An. albimanus* mosquitoes (from the Pacific coast, Mexican gulf and Lacandon Forest lowlands), these two parameters were higher in specimens infected with *P. vivax* Vk210/Pvs25-A *versus* Vk210/Pvs25-B or Vk247/Pvs25-B (*P* < 0.001). For *An. pseudopunctipennis* mosquitoes (from the Pacific coast, northeast Mexico and east Guatemala foothills), the same two parameters were higher in specimens infected with Vk247/Pvs25-B or Vk210/Pvs25-B *versus* Vk210/Pvs25-A (*P* < 0.001). Higher infection rates were caused by Vk247/Pvs25-B than Vk210/Pvs25-B parasites in *An. pseudopunctipennis* (*P* = 0.011*)* and *An. albimanus* (*P* = 0.001). The greatest parasitaemia, gametocytaemia and microgamete formation was observed in Vk247/Pvs25-B infected blood, and each of these parameters correlated with each other and with the number of oocysts in *An. pseudopunctipennis* from the sympatric colony.

**Conclusions:**

*Plasmodium vivax* Vk247/Pvs25-B infections were the most prevalent, likely due to the higher parasitaemia produced in the susceptible vector (especially *An. pseudopunctipennis*). The analysis of mosquito-parasite interactions indicate that *An. pseudopunctipennis* and *An. albimanus* each have a unique pattern of transmitting genetic variants of *P. vivax*, and this is not dependent on geographical origin. The present findings highlight the importance of parasite genotyping to understand transmission dynamics and vectorial participation.

**Electronic supplementary material:**

The online version of this article (10.1186/s13071-019-3331-0) contains supplementary material, which is available to authorized users.

## Background

*Anopheles albimanus* Wiedemann, 1820 and *An. pseudopunctipennis* Theobald, 1901 are the main malaria vectors in Mexico, Central America and northern South America [1]. The former occurs on the coastal plains and in other low-altitude regions from southern USA to northern Peru and the Caribbean Islands [2]. Although no subgroups were revealed by polytene chromosome analysis and cross hybridization [3], differences in allozyme patterns [3] and in ribosomal DNA structure [4] have been documented. *An. pseudopunctipennis* is found at altitudes over 200 m above sea level from southern USA to northern Argentina [5]. Three population clusters have been identified by isozyme analysis, one in southern USA-Guatemala region, another distributed from South America to Central America, and the third in the Antilles [5].

In the last decade, *Plasmodium vivax* has produced more than 99% of malaria cases in Mexico [6, 7]. The known difference between the two *P. vivax* variants lies in the repeat units (Vk) of the circumsporozoite protein (CSP): Vk210 (GRA[A/D]GQPA) [8] and Vk247 (ANGAGNQPG) [9]. Experimental infections using local mosquitoes and infected blood from patients in the Soconusco region (in southern Mexico) showed *An. albimanus* to be more susceptible to Vk210 parasites and *An. pseudopunctipennis* to Vk247 [10]. Variant Vk210 is more prevalent in patients from the coastal plains (where *An. albimanus* predominates), and variant Vk247 in patients from the foothills (where *An. pseudopunctipennis* predominates).

Further studies ascertained the identity and location of the Pvs25 and Pvs28 ookinete surface protein polymorphs. Parasites from the coastal plains were Vk210 with Pvs25 87 Gln/130 Ile and Pvs28 87 Asn/110 Tyr (Pvs25-A/Pvs28-A), identical to those of the Sal I reference strain [11] and infective only for *An. albimanus* [12]. Parasites from the foothills were either Vk210 or Vk247, associated with Pvs25 and displaying substitutions at 130 Thr and Pvs28 87 Asp/110 Asn (Pvs25-B and Pvs28-B), and the Pvs25 substitution 87 Gln-Lys subdivided parasites into B1 or B2. They were more infective for *An. pseudopunctipennis* [12]. An association between cspVk variants of parasites and their local vectors was supported by microsatellite analysis, which provided evidence that *P. vivax* populations are structured according to their sympatric vector distribution. Two related subpopulations were detected in the foothills [13], presumably expressing Pvs25/28 type B [12]. These observations indicate the adaptation and possible selection of parasite variants in each geographical area in accordance with the predominant vector.

The distribution of *An. albimanus* and *An. pseudopunctipennis* along the extensive malarious areas of Mexico follows the pattern reported in the southern part of the State of Chiapas. Although the possible existence of subpopulations of the same mosquito species has not been examined in detail, geographical isolation could favor the selection of some distinguishing characteristics [3, 4]. For instance, there is a different degree of susceptibility to sympatric and allopatric *P. vivax* isolates for *An. albimanus* from El Salvador and Panama [14]. Thus, the association between cspVk/Pvs25 haplotypes and anopheline vectors documented in Chiapas may not occur outside this area.

The aim of the present study was to investigate the susceptibility of *An. albimanus* and *An. pseudopunctipennis*, collected from various geographical sites, to *P. vivax* cspVk/Pvs25 haplotypes from southern Mexico. The results, similar to those previously found in southern Mexico, suggest that distinctive molecular interactions take place between a given parasite haplotype and each mosquito species.

## Methods

### Mosquito collection and colonization

**Fig. 1:**
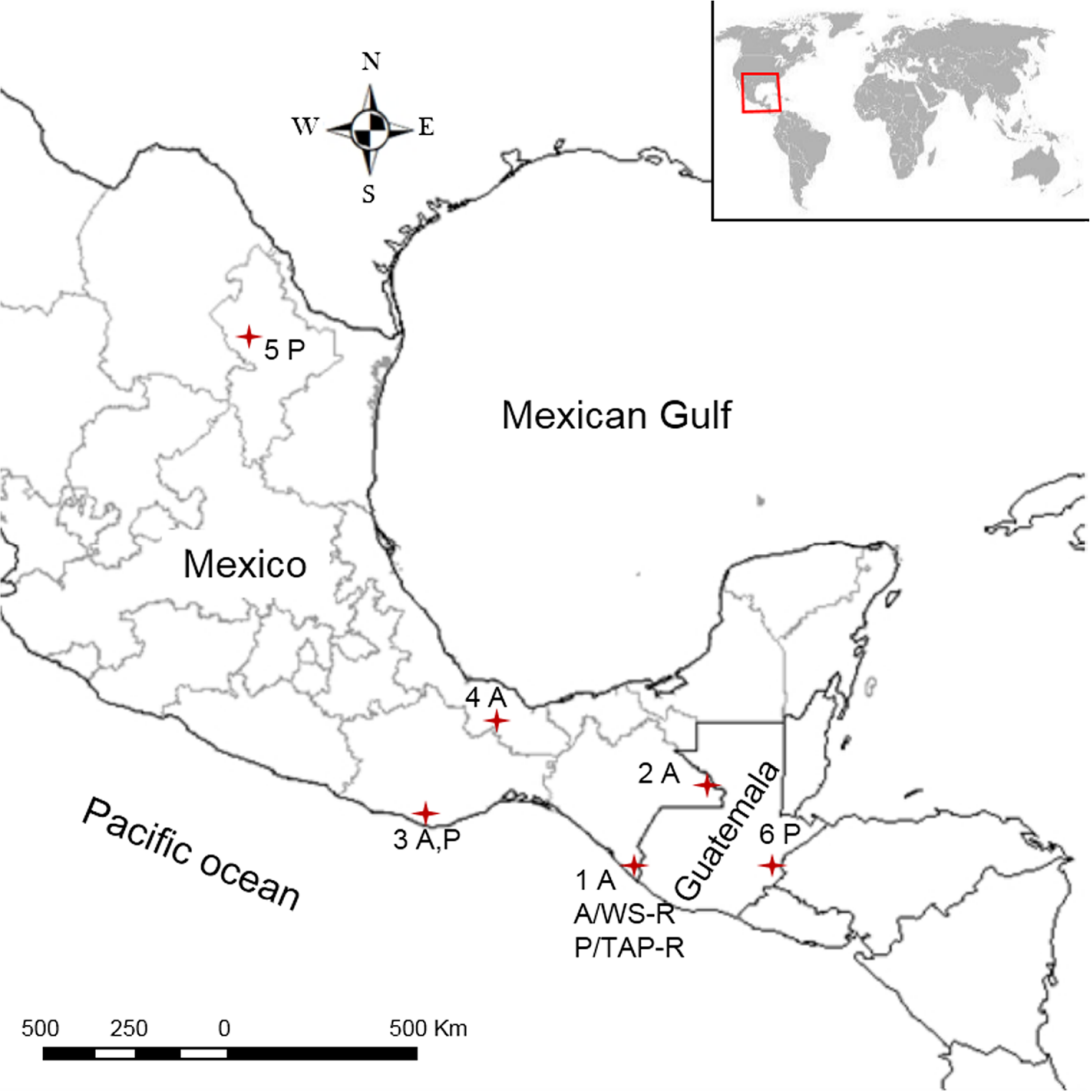
Geographical locations where *Anopheles* mosquitoes were collected. **1** El Encanto (14°59'02.02"N, 92°14'57.11"W), A/WS-R (17°34'23.15"N, 92°00'53.20"W) and P/TAP-R Malpaso (14°59'18.12"N, 92°14'38.70"W), Tapachula, Chiapas. **2** Lacandon Forest, Chiapas (16°31'22.13"N, 90°38'57.17"W). **3** Barra de Colotepec, Oaxaca (15°54'07.08"N, 96°56'16.23"W). **4** Cosamaloapan, Veracruz (18°20'17.45"N, 95°49'37.08"W). **5** Abasolo, Nuevo Leon (25°56'44.47"N, 100°24'31.92"W). **6** Zacapa, Guatemala (14°58'42.93"N, 89°31'39.50"W). *Abbreviations*: A, *An. albimanus*; P, *An. pseudopunctipennis*; A/WS-R, reference strain of *An. albimanus*; P/TAP-R, reference strain of *An. pseudopunctipennis*

Batches of about 750–1300 larvae and/or adult *An. pseudopunctipennis* and/or *An. albimanus* mosquitoes were gathered in Mexico from Lacandon Forest in Chiapas, the Pacific coast regions of the states of Chiapas and Oaxaca, and the Gulf coast areas of the states of Veracruz and Nuevo Leon. Specimens of *An. pseudopunctipennis* were also taken in Zacapa, Guatemala (Fig. 1, Additional file 1: Table S1). In Mexico, the study regions have been historically affected by malaria transmission, but at present malaria is focused on the Pacific coast and along the border with Guatemala [6, 7]. In Guatemala, malaria transmission occurs in most of the country including Zacapa, but the highest number of cases are reported in Escuintla and Alta Verapaz Departments [6].

Adult female mosquitoes were captured while resting in bovine livestock corrals, and larvae were collected from breeding sites with standardized methodologies [15]. Mosquitoes were transported to the Regional Centre for Research in Public Health (CRISP)/INSP for the development of the colonies under insectary conditions. Each batch of larvae and adult mosquitoes was labeled with the date of capture and georeferenced to indicate the town, municipality and state. The species of adult mosquitoes was determined by employing morphological-based keys [16]. Larvae were maintained in trays with water until adult mosquitoes emerged. Adults obtained from larvae were used to establish colonies classified by geographical origin. Mosquito copulation was induced as previously described [17]. Each colony was kept in a different room, and trays were covered with mosquito netting to avoid crossing or contamination between mosquitos from distinct regions. Adult mosquitoes were sustained with cotton impregnated with 5% sugar in water and fed on rabbit blood in artificial membrane feeders. Rabbits were raised and handled according to the Mexican guidelines for laboratory animals [18]. Adult mosquito cages were individually handled to avoid cross contamination between colonies.

The colonies were considered sufficiently stable when natural sexual encounters without any external stimulus resulted in enough females to sustain reproduction (at about the 4th to 16th generations) (Additional file 1: Table S1). Colonies of the white-striped phenotype of *An. albimanus* (A/WS-R) and the Tapachula strain of *An. pseudopunctipennis* (P/TAP-R) served as references [10]. The A/WS-R colony was developed in the late 1990s from mosquitoes gathered in the Soconusco region, the southern-most tip of Chiapas, Mexico. This strain is highly susceptible to *P. vivax* Vk210/Pvs25-A (refers to Pvs25-130Ile) [12, 19] and almost refractory to either *P. vivax* Vk247 or Vk210 and Pvs25-B (refers to Pvs25-130Thr) [12]. The P/TAP-R colony, raised in 1995 from larvae collected in Malpaso (Tapachula, Chiapas) [17], was very susceptible to *P. vivax* Vk247 and Vk210 expressing Pvs25-B but almost resistant to Vk210/Pvs25-A [12]. Groups of 50–200 females (2–5 days post-emergence) of colonized *An. albimanus* and *An. pseudopunctipennis* from the diverse geographical sites were used in susceptibility assays (Additional file 1: Table S1).

### *Plasmodium vivax*-infected blood samples

With prior informed consent, *P. vivax-*infected 5-ml blood samples were obtained from 120 symptomatic patients living on the coast and in the foothills of the Soconusco region. Participating and non-participating patients were treated immediately with chloroquine and primaquine, according to the Mexican guidelines [20].

### Parasitaemia, gametocytaemia and microgamete formation

Microgamete formation was quantified by *in vitro* exflagellation assays (in duplicate) with fresh infected blood. Briefly, immediately after each blood sample was drawn, 10 μl were mixed with 400 μl of cold RPMI medium plus 0.05 mg/ml hypoxanthine at pH 8.3 (Sigma-Aldrich, St. Louis, MO, USA). Ten microliters of each sample was mounted under coverslips and incubated for 15 min at room temperature (about 24 °C) [21]. The total number of rosettes (exflagellations) per preparation were counted under microscope at 400× magnification, and the number of exflagellations per μl was calculated. The complete procedure was accomplished in 30 min. Thick blood smears were prepared and Giemsa stained, as were thin blood smears fixed with methanol. For each sample, the total parasitaemia and gametocytaemia were evaluated by two skilled microscopists who counted fields with up to 500 white blood cells at 1000× magnification. For the calculation of the number of parasites per μl of infected blood, the average number of white cells/μl of blood was considered to be 7000 [22].

### Preparation of *Plasmodium vivax-*infected blood for feeding

Each infected blood sample was centrifuged at 3000 rpm for 5 min and the plasma removed. Blood cells were washed with phosphate buffer at pH 7.2 and 37 °C to delay gamete formation [23] and then centrifuged as above. After discarding the washing solution, blood cells were re-suspended in 40% hematocrit with a non-immune serum of the same blood type, as previously described [10].

### Mosquito feeding, maintenance and infection

The feeding experiments consisted of batches of female mosquitoes of each species from distinct sites and the reference colonies (A/WS-R and P/TAP-R). Female mosquitoes of generations 6–76 (Additional file 1: Table S1) were deprived of sugar-impregnated cotton for 2–6 h before being simultaneously exposed to *P. vivax* infected blood (provided through artificial feeders) for 45–60 min. After the blood-feeding, unfed mosquitoes were removed to maintain only the engorged insects under insectary conditions (24–27 °C, 70–85% humidity). At 7–8 days post-feeding, groups of up to 25 mosquitoes from each experimental colony were examined and their midguts were dissected and treated with mercurochrome to stain the oocysts [10]. The proportion of mosquitoes with at least one oocyst and the number of oocysts per mosquito were recorded.

### *Plasmodium vivax* genotyping

Parasites from blood samples that produced infection in at least one mosquito colony were analyzed for the CSP repeat type and the Pvs25-residue 130 [12]. Briefly, DNA obtained from the infected blood samples was scrutinized for the cspVk by PCR amplification and hybridization, using specific probes as reported [10]. For Pvs25, a gene fragment that had the residue of interest (codon 130) was amplified with primers Pvs25-F23 (nt129–149) 5'-GTG TAT GTG TAA CGA AGG GCT-3' and Pvs25-R214 (nt 453–472) 5'-CAG TTT CTC CCG TTT TGG TA-3'. The amplification mixture contained 100 ng of total genomic DNA, 0.25 mmol of each primer, 1× GoTaq® flexi buffer (pH 8.5), 1.5 mmol MgCl2, 0.6 mmol dNTPs and 1.5 U of GoTaq® Flexi DNA polymerase (Promega, Madison, WI, USA). PCR conditions were: 95 °C for 3 min followed by 35 cycles of 95 °C for 45 s, 60 °C for 45 s, 68 °C for 1 min, and 72 °C for 5 min using a T-100 Thermal Cycler (BioRad, Hercules, CA, USA). Two samples that did not amplify were treated with primers Pvs25-F22 (nt268–289) 5'-ATG TAC AAA TGT GGT TGC ATT G-3' and Pvs25-R214; with these primers the amplification was more efficient. The aforementioned amplification mixture and conditions were employed.

Subsequently, PCR products of the expected molecular size were purified with the Wizard DNA Clean-Up System (Promega) according to the manufacturer’s instructions, and sequenced at the High Throughput Genomics Unit (Department of Genome Sciences, University of Washington, Seattle, WA, USA) or at Macrogen Inc. (Seoul, Republic of Korea). The sequences were aligned by using the homologous sequence of the Sal I genome (XM_001608410.1) and the variation at codon 130 was recorded.

### Statistical analysis

The quantitative variables (i.e. parasitaemia, gametocytaemia and the number of exflagellations) were compared with the Kruskal-Wallis test by ranks [24]. Differences in the proportion of infected mosquitoes were analyzed with logistic regression. The intensity of infection (number of oocyst per infected mosquito) was compared between blood samples with a negative binomial model [25]. These models are suitable for data not showing normal distribution. We assumed that the proportion of infected mosquitoes had a binomial distribution and the oocyst count per infected mosquito a negative binomial distribution. In both models, the species/origin was coded as an 8-level factor. The csp repeat type (Vk210 or Vk247) and Pvs25 residue 130 Ile (Pvs25-A)/Thr (Pvs25-B) were coded as a 3-level factor: Vk247/Pvs25-B, Vk210/Pvs25-A and Vk210/Pvs25-B as reported earlier [12]. The fitted model included all main effects and was examined based on two-way interactions. The statistical significance of the differences was estimated by the linear combination of the regression coefficients, for both the proportion of infected mosquitoes and intensity of infection. The variance of the coefficients was evaluated with the Jackknife variance estimator [26, 27]. A separate logit model was fitted to test the difference in simultaneous infections of *An. pseudopunctipennis* with haplotypes Vk210/Pvs25-B and Vk247/Pvs25-B. The mosquito colonies originating from Guatemala and Nuevo Leon (Mexico) were compared to the reference. The association between parasite blood parameters and the outcome of the susceptible vector was evaluated with Spearman’s rank correlation coefficient [28]. All significance tests were carried out at the 95% confidence level with Stata v.9.2 Statistical Package (Stata Co. 2006).

## Results

### *Plasmodium vivax-*infected blood: parasite counts and haplotypes

In 111 *P. vivax* feeding assays sufficient mosquitoes were fed and survived up to day 7 for midgut inspection, and in 80 of them oocyst infection in at least one mosquito colony was detected. Ten of these resulted from infected blood with haplotype Vk210/Pvs25-A, taken from patients living on coastal plains. Among the other 70 infections, triggered by the blood of patients living in foothills, 16 were of haplotype Vk210/Pvs25-B and 54 of haplotype Vk247/Pvs25-B. Blood infected with Vk247/Pvs25-B had higher parasitaemia than that infected with Vk210/Pvs25-B (Kruskal-Wallis test, *χ*^2^ = 8.42, *df* = 1, *P* = 0.003) and had higher gametocytemia than blood infected with Vk210/Pvs25-B *(*Kruskal-Wallis test, *χ*^2^ = 4.31, *df* = 1, *P* = 0.037) or Vk210/Pvs25-A (Kruskal-Wallis test, *χ*^2^ = 5.23, *df* = 1, *P* = 0.022) (Table 1). No difference in the rate of exflagellation was found between parasite haplotypes (Kruskal-Wallis test, *χ*^2^ = 4.88, *df* = 2, *P* = 0.086).

**Table 1:** *Plasmodium vivax* infected blood: parasitaemia, gametocytaemia and exflagellation rates for each parasite genotype

Blood infection with *P. vivax* genotypes: cspVk/Pvs25-130	*n*	Median (IQR) of parasites/μl of blood	Median (IQR) of gametocytes/μl of blood	*n*	Median (IQR) of exflagellations/μl of blood
Vk210/Pvs25-A^a^	9	5040 (4400–6960)	225 (113–288)	10	36 (12–116)
Vk210/Pvs25-B	16	4360 (2800–6000)	218 (116–628)	16	18 (4–50)
Vk247/Pvs25-B^b^	54	8000 (4640–14,640)	617 (259–1120)	50	42 (20–152)

*Notes*: Parasitaemia was higher with genotype Vk247/Pvs25-B *versus* Vk210/Pvs25-B (*P* = 0.003). Gametocytaemia was higher with genotype Vk247/Pvs25-B *versus* Vk210/Pvs25-A (*P* = 0.022) or Vk210/Pvs25-B (*P* = 0.037). There were no differences in the number of exflagellations between genotypes (*P* = 0.086). Kruskal-Wallis test by ranks (95% CI)

^a^Parasitaemia and gametocytaemia were missing from one sample

^b^The exflagellation values were missing from four samples

*Abbreviation*: *n*, number of parasite isolates

**Fig. 2:**
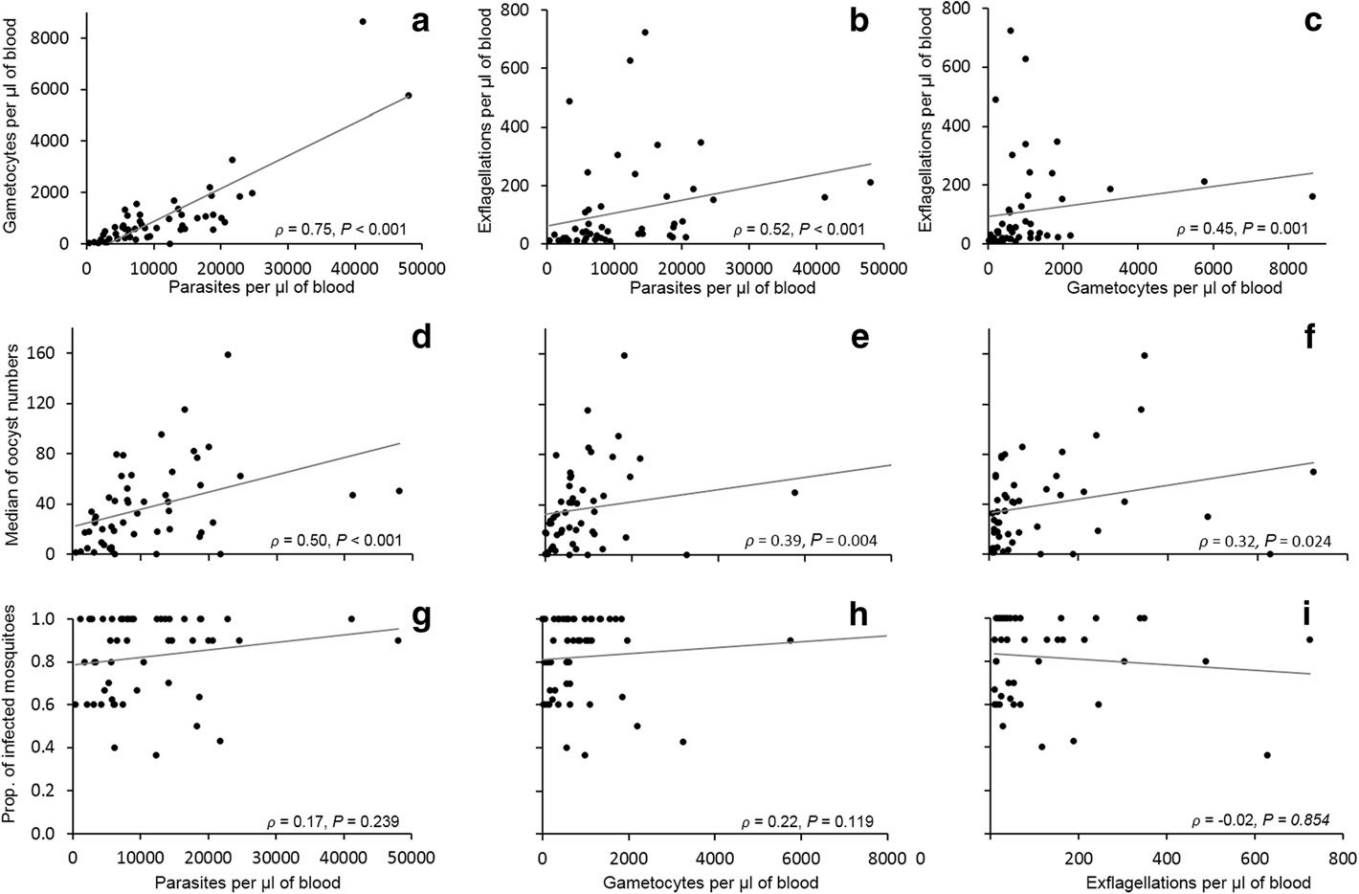
Relationship between *P. vivax* Vk247/Pvs25-B blood parameters and *Anopheles pseudopunctipennis* infection outcomes. Scatter graphs are shown with a regression line. **a-c** The correlation is portrayed between *P. vivax* blood parameters: parasitaemia, gametocytaemia and the number of exflagellations. **d-f** The relation between the data of blood parameters and the median number of oocysts developed in susceptible mosquitoes is depicted. **g-i** The lack of association between blood parameters and the proportion of infected mosquitoes can be appreciated. Spearman’s correlation outputs are indicated in each graph (at 95% CI)

The majority of blood samples were infected with haplotype Vk247/Pvs25-B (67.5%). The 49 experiments that had data from all blood parameters showed that parasitaemia was highly correlated with gametocytaemia (Spearman’s correlation, *ρ* = 0.75, *df* = 48, *P* < 0.001) (Fig. 2a). The correlation of parasitaemia and gametocytaemia with the rate of exflagellation was also significant (Spearman’s correlation: *ρ* = 0.52, *df* = 48, *P* < 0.001 and *ρ* = 0.45, *df* = 48, *P* = 0.001, respectively) (Fig. 2b, c). For Vk210/Pvs25-B (20% of the samples), a correlation was only observed between parasitaemia and gametocytaemia (Spearman’s correlation, *ρ* = 0.75, *df* = 15, *P* = 0.001). For the few Vk210/Pvs25-A samples, no correlation existed between any of the parasite blood parameters.

### Proportion of infection by the *P. vivax* haplotypes in each of the two mosquito species

There was a highly consistent susceptibility pattern for each mosquito species (*An. albimanus* and *An. pseudopunctipennis*) to the different *P. vivax* haplotypes*.* This pattern was independent of the geographical origin of the mosquito colonies.

The proportion of infected *An. albimanus* mosquitoes (from all sites) was 17.35 times higher (95% CI: 12.74–23.64) when fed with blood containing *P. vivax* Vk210/Pvs25-A *versus* Vk210/Pvs25-B or Vk247/Pvs25-B (logistic model Wald Test, *t* = 18.1, *df* = 2247, *P* = 0.001). The proportion of infected mosquitoes from the coast of Chiapas and Veracruz fed with Vk210/Pvs25-B was lower than that of the reference colony (A/WS-R). Similarly, the proportion of infected mosquitoes from Oaxaca and the Lacandon Forest (Chiapas) fed with Vk247/Pvs25-B was lower than that of the reference colony (Table 2).

**Table 2:** The proportion of *An. albimanus* and *An. pseudopunctipennis* mosquitoes (from various geographical sites) infected with oocysts and the corresponding oocyst density produced by different *P. vivax* genotypes (cspVk /Pvs25-130) from southern Mexico

Geographical site and mosquito colony	Vk210/Pvs25-A			Vk210/Pvs25-B			Vk247/Pvs25-B		
	*n*	Prop. infected	Median number of oocysts (IQR)	*n*	Prop. infected	Median number of oocysts (IQR)	*n*	Prop. infected	Median number of oocysts (IQR)
*An. albimanus*									
1. A/WS-R	210	0.72	14 (6.0–28.0)	280	0.10	7 (4.0–17.0)	1133	0.17	4 (1.0–15.0)
1. Coast, Chiapas	–	–	–	65	0.0 ^0.001^	15.0 (15.0–15.0)	70	0.11 ^0.285^	4 (2.5–5.0)
2. LF, Chiapas	10	0.40 ^0.067^	5 (2.5–10.0)	45	0.07 ^0.519^	2.0 (2.0–17.0)	205	0.03 ^<0.001^	9 (3.0–10.0)
3. Oaxaca	10	0.80 ^0.690^	18.5 (7.0–25.0)	56	0.04 ^0.260^	7.0 (6.0–8.0)	119	0.01 ^0.001^	66 (66.0–66.0)
4. Veracruz	10	0.40 ^0.067^	11 (4.0–23.5)	20	0.0	–	35	0.06 ^0.233^	3.5 (1.0–6.0)
*An. pseudopunctipennis*									
1. P/TAP-R	113	0.03	2 (1.0–3.0)	147	0.60	17 (7.0–39.0)	415	0.80	41 (17.0–72.0)
3. Oaxaca	28	0.07 ^0.398^	12.5 (9.0–16.0)	87	0.55 ^0.552^	16.5 (7.0–39.5)	80	0.79 ^0.765^	33 (21.0–57.0)
5. Nuevo Leon	75	0.03 ^0.997^	1 (1.0–1.0)	59	0.53 ^0.391^	19 (8.0–41.0)	275	0.81 ^0.876^	39 (18.0–63.0)
6. Guatemala	68	0.09 ^0.135^	1.5 (1.0–3.0)	100	0.56 ^0.622^	20.5 (8.5–36.5)	207	0.71 ^0.015^	27 (10.5–53.0)^a^

*Notes*: Only infected mosquitoes were considered for the calculation of the median number of oocysts and its IQR. *P*-values from the comparison of the proportion of infected mosquitoes of a colony against the reference strain are indicated as superscripts (underlined are those having significance)

^a^Compared to the reference mosquitoes, the number of oocysts was significantly lower in the Guatemala colony (*P* = 0.002) when fed with parasite genotype Vk247/Pvs25-B. Statistical significance was calculated by using the Jackknife coefficient variance estimator at 95% confidence interval (according to models described in method section)

*Abbreviations*: *n* total number of mosquitoes examined, *Prop*. proportion, *A/WS-R* reference strain of *An. albimanus*, *P/TAP-R* reference strain of *An. pseudopunctipennis*, *LF* Lacandon Forest, *IQR* interquartile range

Likewise, the proportion of infected *An. pseudopunctipennis* mosquitoes (from all sites) was 54.03 times higher (95% CI: 29.94–97.48) when fed with *P. vivax* Vk247/Pvs25-B or Vk210/Pvs25-B *versus* Vk210/Pvs25-A (logistic model Wald test, *t* = 13.26, *df* = 1655, *P* < 0.001). Feeding with *P. vivax* Vk247/Pvs25-B or Vk210/Pvs25-B caused a 19.27 times higher proportion of infected *An. pseudopunctipennis* than infected *An. albimanus* mosquitoes (95% CI: 16.10–23.07; logistic model Wald Test, *t* = 32.25, *df* = 1379, *P* < 0.001). For each parasite haplotype, the proportion of infected mosquitoes in the *An. pseudopunctipennis* colonies (from almost all sites) was similar to that observed in the reference. The only exception was the colony from Guatemala, which had a lower proportion of infected mosquitoes than the reference (logistic model Wald test, *t* = -2.43, *df* = 976, *P* = 0.015) (Table 2).

When only simultaneous infections with the same parasite haplotype were compared to the reference mosquito colony, the proportion of infected individuals was lower in the allopatric mosquito colonies originating from Nuevo Leon and Guatemala (Table 3). For *P. vivax* Vk210/Pvs25-B, the proportion of infected mosquitoes in the Nuevo Leon colony was 0.53 and in the Guatemala colony 0.50, compared to the 0.76 found in P/TAP-R colony (logistic model Wald Test: *t* = -2.23, *df* = 153, *P* = 0.027 and *t* = -2.57, *df* = 153, *P* = 0.011, respectively). Nuevo Leon (Mexico) is about 2000 km from Tapachula, the home of the P/TAP-R colony, and the Guatemala collection site is about 440 km away. There was no difference in the proportion of infection between the P/TAP-R, Guatemala and Oaxaca colonies. For Vk247/Pvs25-B parasites, the only significant difference from P/TAP-R proportion (0.79) was represented by the colony originating from Nuevo Leon (0.65) (logistic model Wald test, *t* = -2.14, *df* = 321, *P* = 0.030) (Table 3).

**Table 3:** Comparison of the mosquitoes infected by different *P. vivax* genotype (cspVk/Pvs25-130) only from simultaneous feeding of *An. pseudopunctipennis* from different geographical sites

Geographical site and mosquito colony	Vk210/Pvs25-B^a^			Vk247/Pvs25-B^b^		
	*n*	Prop. infected	*P*	*n*	Prop. infected	*P*
1. P/TAP-R	49	0.76		107	0.79	
5. Nuevo Leon	49	0.53	0.027	109	0.65	0.030
6. Guatemala	56	0.50	0.011	106	0.72	0.260

*Notes*: Statistical significance was calculated by using the Jackknife coefficient variance estimator at 95% confidence interval

^a^Six simultaneous experiments

^b^Seven simultaneous experiments

*Abbreviations*: *n*, total number of mosquitoes examined; *Prop*., proportion; *P/TAP-R*, reference strain of *An. pseudopunctipennis*

Overall, a 1.4 times higher proportion of both *An. pseudopunctipennis* and *An. albimanus* were infected when fed Vk247/Pvs25-B *versus* Vk210/Pvs25-B parasites (logistic model Wald test, *t* = 4.51, *df* = 3379, *P* < 0.001) (Table 4). Considering only *An. pseudopunctipennis* mosquitoes, the difference in the proportion of infected mosquitos between these two haplotypes ranged from 0.15 to 0.24 (Table 2).

**Table 4:** Differential susceptibility of *An. albimanus* and *An. pseudopunctipennis* mosquitoes to two related *P. vivax* genotypes

*P. vivax* (cspVk/Pvs25-130)	*n*	Prop. infected	Median oocyst number (IQR)
	*An. albimanus*		
Vk210/Pvs25-B	466	0.075	7 (3–17)
Vk247/Pvs25-B	1542	0.132; *P* = 0.001	4 (1–14); *P* = 0.100
	*An. pseudopunctipennis*		
Vk210/Pvs25-B	393	0.56	18 (7–39)
Vk247/Pvs25-B	979	0.784; *P* = 0.011	36 (16–62); *P* < 0.001

*Notes*: The median number of oocysts was calculated only considering the infected mosquitoes. Statistical significance was estimated by using the Jackknife coefficient variance estimator at 95% confidence interval

*Abbreviations*: *n*, total number of mosquitoes examined; *Prop*. proportion, *IQR* interquartile range

### Oocyst density generated by *P. vivax* cspVk/Pvs25-130 haplotypes in *An. albimanus* and *An. pseudopunctipennis*

Comparing only infected *An. albimanus* mosquitoes, a significantly higher oocyst count was found in those infected with the Vk210/Pvs25-A *versus* Vk247/Pvs25-B (negative binomial model Wald test, *t* = 2.99, *df* = 396, *P* = 0.003) (Table 2). Although the number of oocysts was slightly greater in mosquitoes infected with the Vk210/Pvs25-B *versus* Vk247/Pvs25-B or lower with the Vk210/Pvs25-A *versus* Vk210/Pvs25-B haplotypes, the difference was not significant. The few A/WS-R *An. albimanus* infected with Vk210/Pvs25-B or Vk247/Pvs25-B parasites had a small number of oocysts in their midguts. Because the proportion of *An. albimanus* mosquitoes from most other colonies infected with these two parasite haplotypes was also very small, no statistical analysis could be performed.

In assays with *An. pseudopunctipennis*, the intensity of oocyst infection was 8.54 (95% CI: 3.65–19.99) times higher with Vk210/Pvs25-B than with Vk210/Pvs25-A parasites (negative binomial model Wald test, *t* = 4.95, *df* = 1002, *P* < 0.001) and 13.27 (95% CI: 5.73–30.76) times higher when infected with Vk247/Pvs25-B (negative binomial model Wald test, *t* = 6.04, *df* = 1002, *P* < 0.001) (Tables 2, 3). As an exception, two mosquitoes from the Oaxaca colony were infected with the Vk210/Pvs25-A haplotype and had 9 and 16 oocysts in their midgut, respectively. The infected blood used in this assay had the second highest parasitaemia and the highest gametocytaemia and number of rosettes among the Vk210/Pvs25-A parasite lots. The mean number of oocysts was significantly lower in mosquitoes from Guatemala, Nuevo Leon and P/TAP-R colonies with *P. vivax* Vk210/Pvs25-A *versus* Vk247/Pvs25-B or Vk210/Pvs25-B (Table 2). Compared to the reference colony, Vk247/Pvs25-B parasites generated significantly lower oocyst densities in *An. pseudopunctipennis* from Guatemala (negative binomial model Wald test, *t* = -2.99, *df* = 765, *P* = 0.003) (Table 2).

### Correlation between *P. vivax* blood parameters and sympatric mosquito infections

The Vk247/Pvs25-B blood infection parameters correlated with the median number of oocysts in *An. pseudopunctipennis* (P/TAP-R). The number of oocysts showed higher correlation with parasitaemia (Spearman’s correlation, *ρ* = 0.50, *df* = 48, *P* < 0.001) than with gametocytaemia (Spearman’s correlation, *ρ* = 0.39, *df* = 48, *P* = 0.004) or exflagellation rates (Spearman’s correlation, *ρ* = 0.32, *df* = 48, *P* = 0.024) (Fig. 2d, f). In contrast, none of the blood parasite parameters correlated with the proportion of infected mosquitoes (Fig. 2g, i).

Compared to *P. vivax* Vk247/Pvs25-B, fewer assays were conducted with Vk210/Pvs25-B and Vk210/Pvs25-A. With the latter haplotypes, there was no correlation between blood parameters and the median number of oocysts or the proportion of infected mosquitoes, either for *An. pseudopunctipennis* or *An. albimanus*. A low exflagellation rate (*n* = 21, median = 4, IQR = 0–12) was the only blood parasite parameter associated with the absence of oocyst infection in both vector species *versus* the development of oocysts in at least one species (*n* = 80, median = 36, IQR = 14–116) (Kruskal-Wallis test, *χ*^2^ = 26.42, *df* = 1, *P* < 0.001).

## Discussion

The present results confirm our previous findings on different infectivity of *P. vivax* ookinete cspVk/Pvs25 variants for sympatric *An. albimanus* and *An. pseudopunctipennis* in southern Mexico [12]. *Anopheles albimanus* and *An. pseudopunctipennis* mosquitoes from several geographical origins in Mexico (as well as *An. pseudopunctipennis* from nearby Zacapa, Guatemala) were herein exposed to blood with distinct *P. vivax* haplotypes (Vk210/Pv25-A; Vk210/Pvs25-B and Vk247/Pvs25-B). Each of the two mosquito species showed a distinct pattern of susceptibility to the haplotypes, which was consistent for the various geographical locations. Although only parasites from southern Mexico were used to infect mosquitoes, these observations suggest the existence of similar molecular mechanisms for the parasite-mosquito interaction when considering a particular parasite variant and mosquito species from various sites. This evidence points to the probability that parasite variant selection was not the only factor determining the increased infectivity of the parasites to sympatric mosquitoes.

No distinctive populations of *An. albimanus* have been identified, but differences in allozyme patterns [3] and ribosomal DNA structure [4] are indicative of possible early divergence between geographically isolated populations; whereas, three clusters of *An. pseudopunctipennis* were established by isozyme analysis distributed in Mexico, Central and South America [5] and two lineages of *An. pseudopunctipennis* were recently detected in Colombia [29]. Overall, these reports evidence the existence of diverse populations of the two vector species included presently.

Data on the susceptibility of these two anopheline species from different malarious areas is inconclusive. *Anopheles pseudopunctipennis* has been recognized as one of the principal malaria vectors throughout its geographical distribution from Mexico to northern South America [1, 30–33]. However, research on the susceptibility of this anopheline species suggest that it serves as a poor vector for the Central American Sal I and II strains [34]. The involvement of *An. albimanus* in *P. vivax* transmission was first examined decades ago in Central America [14, 35, 36]. Further studies documented that this vector is highly adapted to transmit parasite strains from this region, but showed low susceptibility to *P. vivax* from other geographical sites [37, 38]. Using cspVk variants as markers, field-captured *An. albimanus* displayed infection with *P. vivax* Vk210 and Vk247 at similar rates in Belize [39], but in Colombia, a higher number of sporozoites was detected in the salivary glands of *An. albimanus* when infected with *P. vivax* Vk247 than Vk210 parasites.

Still using cspVk variants as markers, we previously reported differences in southern Mexico between the susceptibility of *An. albimanus* and *An. pseudopunctipennis* to *P. vivax* Vk210 from the coastal regions and to Vk247 from the foothills [10]. We later evidenced that mosquito susceptibility was determined by the capacity of the parasites to invade the midgut epithelium and that ookinetes were unable to invade the midgut epithelium or were destroyed during invasion [40, 41]. Since the production of CSP protein begins in sporoblasts, this molecule plays no role during parasite invasion and the establishment of midgut infections. In further studies, Pvs25 and Pvs28 ookinete surface proteins, conforming haplotypes with cspVk variants, proved to be associated with parasite midgut invasion [12]. The predominant parasite haplotype in coastal areas was Vk210/Pvs25-A, while those in the foothills were Vk210/Pvs25-B and Vk247/Pvs25-B. Parasites with Pvs25-A were more infective for *An. albimanus* and those with Pvs25-B for *An. pseudopunctipennis* [12]. This pattern of susceptibility for these two mosquito species was presently exhibited independently of their geographical origin. However, because of the small numbers of mosquitoes tested, our data are not strictly conclusive but suggest that *An. albimanus* is highly susceptible to *P vivax* Vk210/Pvs25-A in the study sites*.*

Interestingly, higher infection rates were produced by the parasite haplotypes Vk210/Pvs25-B and Vk247/Pvs25-B in sympatric *An. pseudopunctipennis* (P/TAP-R) colony than in the allopatric colonies from Nuevo Leon and Guatemala. These two haplotypes might resemble the foothills population defined by microsatellites (f1/f2) [13]. Although similar recognition molecules were detected in the parasite-mosquito interactions, there may be parasite adaptations and selection driven by local mosquitoes. It is possible that these modifications are engendered by genetic flow and/or evolutionary convergence [42], perhaps a co-adaptation between the cspVk and P25/28 variants and diverse mosquito midgut binding molecules in regions where transmission is carried out by one predominantly abundant vector. As occurs in southern Mexico, the transmission of selective parasite strains by different mosquito species suggests a selective pressure generated by the vector-parasite interaction, as well as geographical isolation caused by the ecological conditions restricting mosquito breeding and distribution [38].

Although susceptibility patterns were similar between colonies of the same mosquito species, the highest infection rates and number of oocysts were produced by Vk247/Pvs25-B parasites in *An. pseudopunctipennis*, which might be related to the high parasitaemia, gametocytaemia and exflagellation rates detected in the blood infected with this *versus* other parasite haplotypes. Similarly, high parasitaemia was observed in patients from Brazil infected with *P. vivax* Vk247 [43]. The present results correspond partially to those reported in India with *An. stephensi* collected in the wild and fed with *P. vivax* infected blood, where the association between parasitaemia and the average number of oocysts was only subtle [44]. Given the low correlation, the degree of infectiveness of gametocytes (difficult to measure) together with certain mosquito factors may influence the establishment of infection and (to some extent) the number of oocysts. The only parameter herein associated with a lack of oocyst development was a low exflagellation rate.

The present findings suggest that distribution and dispersion of *P. vivax* haplotypes might depend on the competence of vector species, which is important for defining control measures. Malaria transmission in Mexico has been reduced to only a few foci and the country is in a pre-elimination stage. In the residual transmission foci, the resilience shown by the parasite in the face of malaria control efforts may owe itself to the high susceptibility of each anopheline species to specific parasite variants, likely similar to the parasite-vector combinations encountered in southern Mexico. The surveillance of possible parasite-reintroductions should be considered.

## Conclusions

*Anopheles albimanus* and *An. pseudopunctipennis*, the main vectors of *P. vivax* in Mexico and Mesoamerica, transmit distinct *P. vivax* haplotypes and with different efficiency. In the various geographical sites herein tested, similarly higher susceptibility was found for *An. albimanus* to *P. vivax* haplotype cspVk/Pvs25-130 Vk210/Pvs25-A than for *An. pseudopunctipennis.* This vector was highly susceptible to haplotypes Vk210/Pvs25-B and Vk247/Pvs25-B. The higher frequency of Vk247/Pvs25-B infections compared to other haplotypes of the parasite is in agreement with its higher parasitaemia and gametocytaemia. The present results also emphasize the importance of genotyping *P. vivax* to understand transmission dynamics and vector participation.

## Additional file

Additional file 1: **Table S1.** *Anopheles albimanus* and *An. pseudopunctipennis*: origin, collection and colonization, and generations used for *P. vivax*-feeding experiments. (DOC 46 kb)

## Abbreviations

INSP: National Institute of Public Health
CRISP: Regional Centre for Research in Public Health
cspVk: circumsporozoite protein central repeat type
WS: white striped phenotype
TAP: Tapachula
A/WS-R: Reference colony of *An. albimanus*
P/TAP-R: Reference colony of *An. pseudopunctipennis*
