# Supplementary material for: Plasmodium vivax CSP-Pvs25 variants from southern Mexico produce distinct patterns of infectivity for Anopheles albimanus versus An. pseudopunctipennis, in each case independent of geographical origin
Source: Parasit Vectors. 2019 Feb 20;12:86. doi: 10.1186/s13071-019-3331-0 (PMC6381756; doi:10.1186/s13071-019-3331-0)
Supplement: Supplementary file 1 — Table S1. Anopheles albimanus and An. pseudopunctipennis: origin, collection and colonization, and generations used for P. vivax-feeding experiments. (DOC 46 kb) [file 13071_2019_3331_MOESM1_ESM.doc]

**Additional file 1**

**Table S1.** *Anopheles albimanus* and *An. pseudopunctipennis*: origin, collection and colonization, and generations used for *P. vivax*-feeding experiments.

| Geographical site^a^ | Locality | State |  | Collections:  Number of specimens and stage | Generations used: | |
| --- | --- | --- | --- | --- | --- | --- |
|  |  |  | Country |  | For colonization^b^ | For experiments^c^ |
| ***An. albimanus*** | |  |  |  |  |  |
| 1 | **A/WS-R (reference)** | Chiapas | Mexico | 850 larvae and 800 adults | 4 | 12-46 |
| 1 | El Encanto (Coast) | Chiapas | Mexico | 760 larvae | 4 | 4-45 |
| 2 | Lacandon forest (LF) | Chiapas | Mexico | 850 adults | 4 | 5-54 |
| 3 | Colotepec | Oaxaca | Mexico | 500 adults | 4 | 6-46 |
| 4 | Cosamaloapan | Veracruz | Mexico | 450 adults | 4 | 5-48 |
|  |  |  |  |  |  |  |
| ***An. pseudopunctipennis*** | |  |  |  |  |  |
| 1 | **P/TAP-R (reference)** | Chiapas | Mexico | 1300 larvae | 6 | 8-46 |
| 3 | Colotepec | Oaxaca | Mexico | 850 larvae and 150 adults | 6 | 8-46 |
| 5 | Abasolo | Nuevo Leon | Mexico | 1200 larvae | 16^d^ | 24–76 |
| 6 | - | Zacapa | Guatemala | 1300 larvae | 6 | 8-46 |

^a^See Figure 1

^b^Number of generations to consider a colony established

^c^Number of generations used to feed with *P. vivax* infected bloods and in here reported

^d^This colony required long time to establish, probably due to its origin; alike other colonies, specimens were collected in the Nearctic region, with arid and semi-arid climate presenting high variation in temperature, humidity and photoperiod during the year
